# Cross-Sectional and Longitudinal Associations of Different Sedentary Behaviors with Cognitive Performance in Older Adults

**DOI:** 10.1371/journal.pone.0047831

**Published:** 2012-10-17

**Authors:** Emmanuelle Kesse-Guyot, Hélène Charreire, Valentina A. Andreeva, Mathilde Touvier, Serge Hercberg, Pilar Galan, Jean-Michel Oppert

**Affiliations:** 1 UMR Inserm U557, Inra U1125, Cnam, University Paris 13 Paris-cité-Sorbonne, Centre of Research on Human Nutrition Ile-de-France, Bobigny, France; 2 Paris-Est Créteil University, Department of Geography, Lab-Urba, Urbanism Institute of Paris, Paris, France; 3 Department of Public Health, Hôpital Avicenne, Bobigny, France; 4 Université Pierre et Marie Curie-Paris 6, Dept of Nutrition Pitié-Salpêtrière Hospital (AP-HP), Centre of Research on Human Nutrition Ile-de-France, Paris, France; Oregon Health & Science University, United States of America

## Abstract

**Background:**

The deleterious health effects of sedentary behaviors, independent of physical activity, are increasingly being recognized. However, associations with cognitive performance are not known.

**Purpose:**

To estimate the associations between different sedentary behaviors and cognitive performance in healthy older adults.

**Methods:**

Computer use, time spent watching television (TV), time spent reading and habitual physical activity levels were self-reported twice (in 2001 and 2007) by participants in the SUpplémentation en Vitamines et MinérauX (SU.VI.MAX and SU.VI.MAX2) study. Cognitive performance was assessed at follow-up (in 2007–2009) via a battery of 6 neuropsychological tests used to derive verbal memory and executive functioning scores. Analyses (ANCOVA) were performed among 1425 men and 1154 women aged 65.6±4.5 at the time of the neuropsychological evaluation. We estimated mean differences with 95% confidence intervals (95%CI) in cognitive performance across categories of each type of sedentary behavior.

**Results:**

In multivariable cross-sectional models, compared to non-users, participants using the computer for >1 h/day displayed better verbal memory (mean difference = 1.86; 95%CI: 0.95, 2.77) and executive functioning (mean difference = 2.15; 95%CI: 1.22, 3.08). A negative association was also observed between TV viewing and executive functioning. Additionally, participants who increased their computer use by more than 30 min between 2001 and 2007 showed better performance on both verbal memory (mean difference = 1.41; 95%CI: 0.55, 2.27) and executive functioning (mean difference = 1.41; 95%CI: 0.53, 2.28) compared to those who decreased their computer use during that period.

**Conclusion:**

Specific sedentary behaviors are differentially associated with cognitive performance. In contrast to TV viewing, regular computer use may help maintain cognitive function during the aging process.

**Clinical Trial Registration:**

clinicaltrial.gov (number NCT00272428).

## Introduction

Due to the overall aging of the population, a dramatic increase in age-related diseases is expected in the years to come, pointing to the need for prevention efforts capitalizing on environmental and behavioral strategies [1]–[3]. Findings from both observational and intervention studies are in favor of a beneficial effect of physical activity on preventing cognitive decline or the development of dementia [4]–[6]. However, most prevention-focused epidemiologic studies pertain to elderly or very old populations and there is a gap in knowledge of preclinical middle-age determinants. Current knowledge postulates that cognitive decline leading to Alzheimer's disease begins relatively early in life and progresses insidiously for decades before it is clinically expressed [7], arguing for the need to identify midlife factors associated with cognitive aging [8], [9].

Parallel with physical activity, there is increasing interest in sedentary behavior as a complementary determinant of health outcomes [10]–[15]. Sedentary behavior refers to activities that do not increase energy expenditure above the resting level [10], [16]. In a recent meta-analysis, television (TV) viewing, a typical sedentary behavior, was shown to be associated with increased risk of type 2 diabetes, cardiovascular disease and all-cause mortality [17], independent of habitual physical activity levels. Less is known about the health effects of other sedentary behaviors such as reading or computer use [11]. In addition, compared to the available evidence regarding sociodemographic and behavioral correlates of sedentary behaviors, only limited research has been conducted on the relationship between sedentary behaviors and cognitive outcomes [11]. Better knowledge of these associations is needed for identifying at-risk groups and for launching preventive actions [11].

Some studies point out the potential beneficial effect of engaging in cognitively stimulating leisure activities on cognitive aging [18]–[31]. However, most of these studies have used composite frequency measure without considering the sedentary nature of such activities [18], [21]–[27], [29]–[31].

The aim of the present study was to assess whether different sedentary behaviors, including TV viewing, computer use and reading, would be differentially associated with indicators of cognitive function in healthy older adults.

## Materials and Methods

### Study population

The SU.VI.MAX (SUpplémentation en VItamines et Minéraux AntioXydants) study was a population-based, double-blind, placebo-controlled, randomized trial assessing the efficacy of a daily antioxidant supplementation on the incidence of cardiovascular disease and cancer [32], [33]. The trial was launched in 1994–95 with a planned follow-up of 8 years (1994–2002). From the full SU.VI.MAX cohort (N = 13,017), a total of 6,850 participants who had agreed to participate in a post-supplementation observational follow-up were recruited for the SU.VI.MAX 2 study (2007–2009) which included a neuropsychological evaluation [34].

### Ethics Statement

The SU.VI.MAX and SU.VI.MAX 2 studies were conducted according to the guidelines laid down in the Declaration of Helsinki and were approved by the Ethics Committee for Studies with Human Subjects of the Paris-Cochin Hospital (CCPPRB n° 706 and n° 2364, respectively) and the *Comité National Informatique et Liberté* (CNIL n° 334641 and n°907094, respectively). Written informed consent was obtained from all participants.

### Inclusion criteria

Among the 6,850 adults included in the SU.VI.MAX 2 study (2007−2009), all cognitive tests were completed by a total of 4,447 individuals who were aged 45–60 y at the start of the SU.VI.MAX trial in 1994. Among them, 2,835 participants had available data on sedentary behavior and physical activity from both 2001 and 2007. From that subsample, a total of 2,612 participants had available data for all covariables of interest. Finally, 33 participants who had been confined to bed for more than 1 month during the period covered by the physical activity questionnaires were excluded, leaving 2,579 participants for inclusion in the present analyses.

### Cognitive functioning assessment

At the SU.VI.MAX 2 phase, the participants were invited to take part in a neuropsychological evaluation using validated tests regarding three memory domains (lexical-semantic, episodic and working) and mental flexibility. Lexical-semantic memory was assessed by verbal fluency tasks, including a phonemic fluency task (citing words beginning with the letter P) and a semantic fluency task (naming as many animals as possible). The score for each task was the number of correct words produced during a 2-min period [35]. Episodic memory was evaluated using the RI-48 test, which is a delayed cued recall test with a maximum score of 48 [36]. Working memory was assessed with the forward and backward digit span. One point was scored for each sequence repeated correctly, with a maximum score of 14 points for digit span forward as well as backward [37]. Mental flexibility was assessed through the Delis-Kaplan trail-making test (TMT), connecting numbers and letters alternating between the two series. The score was the time in seconds needed to complete the task [38].

### Sedentary behaviors and physical activity

Sedentary behaviors and physical activity were assessed in 2001 and 2007 using a self-administered French version of the Modifiable Activity Questionnaire (MAQ) [39], [40].

The original MAQ was designed to assess sedentary and physical activity during both leisure time and work over the past 12 months [41] and was the instrument used in the Diabetes Prevention Program [42]. The MAQ has been validated against energy expenditure measurements using the doubly-labeled water technique, and the test-retest properties of the questionnaire have been established [43]. Generally, participants were asked to report each leisure-time physical activity performed at least 10 times for at least 10 min per session over the past 12 months. The frequency and duration of each activity were also reported. For each reported physical activity, the number of hours per week was multiplied by its estimated metabolic cost expressed in metabolic equivalent tasks (MET) [44]. Then, a summary score was obtained expressed in MET-h per week. In the same questionnaire, participants were asked to report their average daily time spent watching TV, using a computer, or reading, and each of these variables was expressed in min per day.

### Covariates

At the start of the SU.VI.MAX trial, information on gender, date of birth, education and social position was collected. Retirement status was assessed at the time of each MAQ administration. At the SU.VI.MAX 2 phase, depressive symptoms were assessed using the French version of the Center for Epidemiologic Studies Depression Scale (CES-D) and the score was modeled as a covariate [45]. Tobacco use status (never, former, current smoker) and self-rated health status were collected through a self-administered questionnaire. Self-rated health status was assessed on a 5-point scale (excellent, good, fair, poor, very poor). During the clinical examination, weight was measured using an electronic scale, with participants wearing indoor clothing and no shoes. Height was measured under the same conditions with an electronic wall-mounted stadiometer. During the entire follow-up (1994–2009), the incidence of cardiovascular disease, hypertension and diabetes was documented [46].

### Statistical analysis

Principal component analysis (PCA) was performed in order to yield summary measures accounting for the correlations among the cognitive scores, thereby maximizing the explained variance [47]. These summary scores were converted into T scores (mean = 50, SD = 10). Thus, a one-point difference in the test score corresponded to one-tenth of a SD difference. For each sedentary behavior, the original values (duration expressed in min/day) and change over time were categorized in three classes based on the respective distribution (tertiles or none/low/ high according to the median value). Body mass index (BMI) was calculated as the ratio of weight to squared height (kg/m^2^). Time-dependent retirement status was computed as follows: retired at baseline, retired during follow-up, not retired at the end of follow-up.

Descriptive characteristics of the study sample are reported as mean (SD) or percentage. From the 13,017 participants included in SU.VI.MAX, those retained and those excluded from the present analyses were compared in order to assess potential selection bias. Reported P-values refer to the chi^2^ test or Wilcoxon-Mann-Whitney test, as appropriate. Covariance analyses were used to estimate the difference in mean (95% confidence interval, CI) cognitive scores across categories of sedentary behaviors as well as the 6-year change in sedentary behaviors using the lowest category as reference. P for trend was assessed, using linear contrast tests across categories. The initial model was adjusted only for the interval between sedentary behavior assessment and cognitive evaluation. The second model was adjusted for age (y), education (primary, secondary, university or equivalent), supplementation group (active or placebo), BMI, occupational category (unemployed, manual workers, employed, self-employed or farmers, managerial staff) and retirement status (yes/no). The third model was further adjusted for tobacco use status, BMI, depressive symptoms, history of hypertension (yes/no), history of diabetes (yes/no), and history of cardiovascular diseases (yes/no). The full final model was further adjusted for leisure-time physical activity (MET-h/week) and the remaining sedentary behaviors. The longitudinal models were similarly conducted except for an adjustment for the baseline score for each sedentary behavior. Retirement was considered a time-dependent covariable and the remaining sedentary behaviors (ie, those not modeled as the respective independent variable) in the full model were accounted for as change over time.

In an effort to assess the robustness of our results, we carried out sensitivity analyses after removing participants with the lowest cognitive performance scores (eg, below the education level-specific tenth percentile), as these participants may have modified their physical activity or sedentary behaviors following changes in cognitive abilities. Interaction terms with gender and retirement status regarding sedentary behaviors were tested.

All tests were two-sided and type I error was set at 5%. Statistical analyses were performed using SAS software (version 9.1, SAS Institute Inc, Cary, NC, USA).

## Results

### Participants characteristics

Baseline and follow-up descriptive characteristics of the participants included in the present study are presented in Table 1. At the time of the cognitive assessment, mean age of the 2,179 included participants (1,425 men and 1,154 women) was 65.6 (SD = 4.5) y and 55% were male. More than two thirds of the participants rated their health as “good” or better. Few of the participants were smokers and the majority was retired.

**Table 1:** Baseline and follow-up sociodemographic and behavioral characteristics of the participants (N = 2,579) a.

	Mean (SD) or %
Male, %	55.3
Age (1994), y a	52.2 (4.6)
Age (2007–2009), y b	65.6 (4.5)
BMI, kg/m^2^ b	25.5 (3.8)
Intervention group, %	52.6
Education, % a	
Primary	19.9
Secondary	40.7
University	39.4
Smoking status, % b	
Never-smokers	45.9
Former smokers	43.4
Current smokers	10.6
History of diabetes, %	6.8
History of hypertension, %	58.7
History of cardiovascular disease, %	4.7
Retired, % b	84.5
Depression score (CES-D) b	8.5 (7.3)
Leisure-time physical activity^,^ MET-h/week b	26.5 (27.2)
Watching TV, min/d b	147.2 (82.9)
Computer use, min/d b	48.8 (61.6)
Reading, min/d b	73.0 (54.9)
General health status, % a	
Excellent	10.4
Good	69.0
Fair	19.4
Poor	1.2
Very poor	0.04

CES-D, Center for Epidemiologic Studies Depression Scale.

a At baseline (1994).

b At the SU.VI.MAX 2 examination (2007–2009).

Comparisons between included and excluded participants are shown (Table S1). Compared to included participants, those SU.VI.MAX participants who were excluded from the present analysis were younger, and therefore less likely to be retired, more often female (reflecting the initial SU.VI.MAX design) and smokers. They were also more active and spent more time engaged in sedentary behaviors. They also reported poorer general health and were more likely to report a history of diabetes and current depressive symptoms.

### Cognitive factor identification

Two major cognitive patterns were extracted with PCA, accounting for 61% of the total initial variance. The first factor reflected semantic (factor loading = 0.80) and phonemic fluency (factor loading = 0.65) and the RI-48 cued recall task (factor loading = 0.76), and it accounted for 42% of the total variance. The second factor accounted for 19% of the total variance and reflected the forward (factor loading = 0.85) and backward (factor loading = 0.83) digit span tasks and, to a lesser extent, the TMT (factor loading = 0.49). These two factors were interpreted as reflecting “verbal memory” and “executive functioning,” respectively, and were used in the subsequent analyses as the main variables regarding cognitive performance.

### Sedentary behaviors and cognitive performance

No interaction between any of the sedentary behaviors and gender were detected (all P>0.05) regarding cognitive function, thus analyses were conducted in the full sample. Cross-sectional associations between categories of the different sedentary behaviors (computer use, watching TV and reading) and indicators of cognitive performance are presented in Table 2. More time spent using the computer was associated with both better verbal memory and better executive functioning in crude and adjusted models. In the crude model, time spent watching TV was negatively associated with performance on both verbal memory and executive functioning. In the fully-adjusted cross-sectional model, more time spent watching TV remained associated with lower executive functioning scores. Reading was not associated with verbal memory. In addition, the association between more time spent reading and lower executive functioning scores did not remain significant in the fully adjusted models.

**Table 2:** Associations between time spent in sedentary behaviors (min/day) and cognitive function (N = 2,579) a.

		Verbal memory			Executive functioning		
Sedentary behavior		T2	T3	P^b^	T2	T3	P^b^
Computer use	Model a^c^	2.96 (2.00–3.92)	2.51 (1.59–3.44)	<0.0001	2.12 (1.16–3.08)	3.67 (2.75–4.59)	<0.0001
	Model b^d^	1.63 (0.70–2.56)	1.91 (1.00–2.81)	<0.0001	0.64 (−0.31–1.60)	2.22 (1.30–3.15)	<0.0001
	Model c^e^	1.58 (0.64–2.52)	1.86 (0.95–2.77)	<0.0001	0.60 (−0.36–1.56)	2.16 (1.23–3.09)	<0.0001
	Model d^f^	1.53 (0.58–2.48)	1.86 (0.95–2.77)	<0.0001	0.47 (−0.49–1.44)	2.15 (1.22–3.08)	<0.0001
TV viewing	Model a^c^	−0.79 (−1.76–0.18)	−1.49 (−2.42– −0.56)	0.002	−1.19 (−2.15– −0.22)	−2.27 (−3.19– −1.34)	<0.0001
	Model b^d^	−0.34 (−1.25–0.58)	−0.10 (−1.00–0.79)	0.82	−0.63 (−1.56–0.31)	−1.03 (−1.94– −0.11)	0.03
	Model c^e^	−0.28 (−1.20–0.63)	−0.05 (−0.95–0.86)	0.92	−0.57 (−1.50–0.37)	−0.99 (−1.92– −0.06)	0.04
	Model d^f^	−0.28 (−1.20–0.64)	−0.06 (−0.97–0.84)	0.89	−0.50 (−1.43–0.44)	−1.02 (−1.95– −0.10)	0.03
Reading	Model a^c^	0.73 (−0.21–1.68)	0.57 (−0.37–1.52)	0.23	−0.76 (−1.71–0.18)	−0.96 (−1.90– −0.01)	0.05
	Model b^d^	0.56 (−0.33–1.45)	0.30 (−0.61–1.20)	0.52	−0.84 (−1.75–0.07)	−0.88 (−1.81–0.05)	0.06
	Model c^e^	0.57 (−0.32–1.47)	0.26 (−0.65–1.17)	0.57	−0.85 (−1.76–0.06)	−0.95 (−1.88– −0.02)	0.05
	Model d^f^	0.54 (−0.35–1.43)	0.23 (−0.68–1.14)	0.62	−0.82 (−1.73–0.09)	−0.89 (−1.81–0.04)	0.06

T2, T3: tertile of time spent in each of the sedentary behaviors.

Tertile 1 used as reference.

a Values are mean difference (95% confidence interval) in cognitive scores, lowest category as reference.

b P for trend across categories.

c model a (crude): interval between sedentary behavior assessment and cognitive evaluation.

d model b: model a + age, gender, supplementation group, education, occupational categories, retirement status.

e model c: model b + tobacco use status, BMI, CES-D score, general health status, history of cardiovascular diseases, diabetes and hypertension.

f model d: model c + leisure-time physical activity, remaining sedentary behaviors (TV, reading, computer according to main exposure).

### Cross-time change in sedentary behaviors and cognitive performance

The associations between the 6-year change in each sedentary behavior and cognitive function at follow-up are shown in Table 3. The increase in time spent using the computer was associated with better verbal memory and executive functioning, in crude and adjusted models. Changes in time spent watching TV or time spent reading were not associated with subsequent cognitive performance.

**Table 3:** Associations between change in sedentary behaviors over 6 years and cognitive function (N = 2,579) a.

		Verbal memory			Executive functioning		
Change in sedentary behavior		T2	T3	P^b^	T2	T3	P^b^
Δ Computer use	Model a^c^	2.57 (1.59–3.55)	1.93 (1.04–2.82)	<0.0001	2.16 (1.18–3.14)	2.46 (1.57–3.34)	<0.0001
	Model b^d^	1.42 (0.48–2.37)	1.47 (0.62–2.32)	0.001	1.10 (0.13–2.06)	1.44 (0.56–2.31)	0.001
	Model c^e^	1.40 (0.45–2.34)	1.41 (0.56–2.27)	0.001	1.06 (0.10–2.03)	1.39 (0.52–2.27)	0.002
	Model d^f^	1.34 (0.38–2.29)	1.41 (0.55–2.27)	0.001	0.95 (−0.02–1.92)	1.41 (0.53–2.28)	0.002
Δ TV viewing	Model a^c^	0.26 (−0.67–1.18)	−0.65 (−1.58–0.27)	0.17	0.56 (−0.38–1.51)	−0.11 (−1.06–0.84)	0.82
	Model b^d^	0.27 (−0.66–1.19)	−0.65 (−1.59–0.28)	0.17	0.10 (−0.84–1.05)	−0.32 (−1.27–0.63)	0.50
	Model c^e^	0.31 (−0.62–1.24)	−0.64 (−1.58–0.30)	0.18	0.17 (−0.78–1.11)	−0.30 (−1.25–0.66)	0.54
	Model d^f^	0.81 (−0.13–1.76)	0.27 (−0.68–1.21)	0.58	0.21 (−0.73–1.15)	−0.36 (−1.32–0.59)	0.46
Δ Reading	Model a^c^	−0.04 (−0.99–0.90)	−0.07 (−1.02–0.88)	0.89	0.14 (−0.80–1.09)	−0.16 (−1.11–0.79)	0.74
	Model b^d^	0.13 (−0.81–1.08)	0.11 (−0.83–1.05)	0.82	−0.41 (−1.38–0.56)	−0.47 (−1.43–0.49)	0.34
	Model c^e^	0.13 (−0.82–1.08)	0.06 (−0.89–1.00)	0.91	−0.41 (−1.38–0.56)	−0.59 (−1.56–0.37)	0.23
	Model d^f^	0.13 (−0.82–1.08)	0.04 (−0.92–0.99)	0.94	−0.36 (−1.33–0.60)	−0.61 (−1.57–0.36)	0.22

T2, T3: tertile of change in time spent in each of the sedentary behaviors.

Tertile 1 used as reference.

a Values are mean difference (95% confidence interval) in cognitive scores, lowest category as reference.

b P for trend across categories.

c model a (crude): interval between first sedentary behavior assessment and cognitive evaluation.

d model b: model a + age, gender, supplementation group, education, occupational categories, time-dependent retirement status + baseline value.

e model c: model b + tobacco use status, BMI, CES-D score, general health status, history of cardiovascular diseases, diabetes and hypertension.

f model d: model c + delta of leisure-time physical activity, delta of remaining sedentary behaviors.

### Sensitivity analyses

Cross-sectional and longitudinal analyses performed in a sub-sample (N = 2,321) after removing participants with the lowest cognitive performance scores (eg, below the education level-specific tenth percentile) showed similar, although weaker, associations (Table 4 and Table 5). The only notable difference was that the relationship between change in time spent using the computer and verbal memory performance was no longer detectable.

**Table 4:** Cross-sectional associations between engagement in sedentary behaviors (min/day) and cognitive function after removing participants with the lowest cognitive performance scores (eg, below the education level-specific tenth percentile) (N = 2,321) a.

		Verbal memory			Executive functioning		
Sedentary behavior		T2	T3	P^b^	T2	T3	P^b^
Computer use	Model a^c^	2.01 (1.05–2.98)	1.44 (0.52–2.37)	0.002	1.77 (0.77–2.76)	3.03 (2.08–3.98)	<0.0001
	Model b^d^	0.78 (−0.16–1.71)	0.86 (−0.04–1.77)	0.06	0.29 (−0.70–1.27)	1.52 (0.57–2.47)	0.002
	Model c^e^	0.78 (−0.16–1.72)	0.82 (−0.09–1.73)	0.08	0.25 (−0.73–1.24)	1.45 (0.49–2.41)	0.003
	Model d^f^	0.78 (−0.17–1.72)	0.83 (−0.09–1.74)	0.08	0.17 (−0.83–1.16)	1.44 (0.48–2.40)	0.003
TV viewing	Model a^c^	−0.74 (−1.70–0.22)	−1.29 (−2.21–0.37)	0.01	−1.06 (−2.05–0.08)	−2.23 (−3.18–1.29)	<0.0001
	Model b^d^	−0.21 (−1.11–0.69)	0.12 (−0.77–1.01)	0.79	−0.44 (−1.39–0.51)	−0.96 (−1.89–0.02)	0.05
	Model c^e^	−0.19 (−1.10–0.72)	0.09 (−0.81–0.99)	0.84	−0.40 (−1.35–0.56)	−0.96 (−1.91–0.01)	0.05
	Model d^f^	−0.20 (−1.11–0.70)	0.08 (−0.82–0.98)	0.87	−0.35 (−1.30–0.60)	−0.98 (−1.93–0.04)	0.04
Reading	Model a^c^	1.29 (0.36–2.22)	1.14 (0.21–2.08)	0.02	−0.46 (−1.42–0.50)	−0.58 (−1.54–0.39)	0.24
	Model b^d^	1.06 (0.19–1.94)	0.81 (−0.09–1.70)	0.08	−0.52 (−1.45–0.41)	−0.51 (−1.46–0.43)	0.29
	Model c^e^	−0.52 (−1.45–0.41)	−0.57 (−1.52–0.37)	0.24	1.07 (0.19–1.95)	0.75 (−0.15–1.65)	0.10
	Model d^f^	−0.52 (−1.44–0.41)	−0.56 (−1.51–0.38)	0.24	1.04 (0.16–1.92)	0.71 (−0.19–1.61)	0.12

T2, T3: tertile of time spent in each of the sedentary behaviors.

Tertile 1 used as reference.

a Values are mean difference (95% confidence interval) in cognitive scores, lowest category as reference.

b P for trend across categories.

c model a (crude): interval between sedentary behavior assessment and cognitive evaluation.

d model b: model a + age, gender, supplementation group, education, occupational categories, retirement status.

e model c: model b + tobacco use status, BMI, CES-D score, general health status, history of cardiovascular diseases, diabetes and hypertension.

f model d: model c + leisure-time physical activity, remaining sedentary behaviors (TV, reading, computer according to main exposure).

**Table 5:** Longitudinal associations between change in sedentary behaviors over 6 years and cognitive function after removing participants with the lowest cognitive performance scores (eg, below the education level-specific tenth percentile) (N = 2,321)^a^.

		Verbal memory			Executive functioning		
Change in sedentary behavior		T2	T3	P^b^	T2	T3	P^b^
Δ Computer use	Model a^c^	1.83 (0.85–2.80)	0.95 (0.07–1.83)	0.03	1.91 (0.90–2.91)	1.90 (1.00–2.81)	<0.0001
	Model b^d^	0.69 (−0.24–1.63)	0.56 (−0.28–1.41)	0.19	0.85 (−0.14–1.84)	0.88 (−0.01–1.77)	0.05
	Model c^e^	0.72 (−0.22–1.66)	0.52 (−0.33–1.37)	0.23	0.83 (−0.16–1.82)	0.82 (−0.07–1.72)	0.07
	Model d^f^	0.69 (−0.25–1.64)	0.52 (−0.33–1.38)	0.23	0.75 (−0.25–1.74)	0.83 (−0.06–1.73)	0.07
Δ TV viewing	Model a^c^	0.81 (−0.12–1.75)	0.05 (−0.90–1.00)	0.91	0.27 (−0.69–1.23)	0.06 (−0.91–1.04)	0.90
	Model b^d^	0.48 (−0.43–1.39)	−0.17 (−1.09–0.75)	0.71	−0.09 (−1.05–0.87)	−0.03 (−1.01–0.94)	0.95
	Model c^e^	0.47 (−0.45–1.38)	−0.21 (−1.14–0.72)	0.66	−0.05 (−1.01–0.91)	−0.03 (−1.01–0.94)	0.95
	Model d^f^	0.50 (−0.42–1.42)	−0.19 (−1.12–0.75)	0.69	−0.01 (−0.97–0.95)	−0.11 (−1.09–0.87)	0.83
Δ Reading	Model a^c^	0.11 (−0.83–1.05)	0.12 (−0.82–1.05)	0.81	0.29 (−0.68–1.25)	0.01 (−0.96–0.97)	0.99
	Model b^d^	0.62 (−0.31–1.56)	0.57 (−0.36–1.50)	0.23	−0.13 (−1.12–0.86)	−0.15 (−1.13–0.83)	0.76
	Model c^e^	0.62 (−0.32–1.56)	0.50 (−0.43–1.43)	0.29	−0.12 (−1.10–0.87)	−0.27 (−1.25–0.72)	0.60
	Model d^f^	0.61 (−0.33–1.55)	0.47 (−0.47–1.41)	0.33	−0.11 (−1.10–0.88)	−0.31 (−1.30–0.67)	0.54

T2, T3: tertile of change in time spent in each of the sedentary behaviors.

Tertile 1 used as reference.

a Values are mean difference (95% confidence interval) in cognitive scores, lowest category as reference.

b P for trend across categories.

c model a (crude): interval between first sedentary behavior assessment and cognitive evaluation.

d model b: model a + age, gender, supplementation group, education, occupational categories, time-dependent retirement status + baseline value.

e model c: model b + tobacco use status, BMI, CES-D score, general health status, history of cardiovascular diseases, diabetes and hypertension.

f model d: model c + delta of leisure-time physical activity, delta of remaining sedentary behaviors.

## Discussion

In this population of older French adults, computer use was positively associated with a better performance in different cognitive domains, i.e. verbal memory and executive functioning, after controlling for a number of covariates including physical activity. In addition, the increase in time spent using the computer over 6 years was associated with better cognitive performance in both domains. A negative cross-sectional association was observed between TV viewing and executive functioning, which did not persist in the longitudinal models.

Our study provides new insights on the association between specific sedentary behaviors and cognitive function in healthy older adults.

### Strengths

Some strengths of the study should be noted. In this population of aging adults, engagement in specific types of sedentary behavior was assessed at two time points using the same instrument. In turn, we employed a battery of sensitive neuropsychological tests known to limit floor or ceiling effects. Although residual confounding cannot be ruled out in observational studies, we were able to take into account a number of potential confounders, including educational level, health status, and presence of depressive symptoms, all of which have been found to be associated with sedentary behavior [11]. Finally, the present analyses included equal proportions of men and women.

### Sedentary behaviors and cognitive function

A major finding of this study was the positive cross-sectional and longitudinal associations of the two dimensions of cognitive function with time spent using a computer. In turn, a negative (in cross-sectional models) or non-significant (in longitudinal models) relationship was found between cognitive function and TV viewing. The findings add to the presently scant literature on computer use and cognitive performance [48], [49]. In fact, we could find no prior studies assessing both constructs simultaneously. In one previous US study [48], better cognitive performance, especially executive function, was observed among frequent computer users in a strictly cross-sectional design with adults aged 32–84 years. However, in that study, the authors had assessed the frequency but not the average daily duration of computer use. In an earlier US report in adults [49], cognitive abilities (assessed through a standardized battery of 21 measures) were associated with computer use, but neither the frequency nor duration of use was specified. In the present study, sensitivity analyses showed that the association between computer use and verbal memory did not persist once individuals with low cognitive function scores were removed, arguing for further investigations regarding specific cognitive domains.

In most previous studies, sedentary behaviors have been grouped according to whether they were mainly mental, physical or social. For example, data on TV viewing have been previously modeled in a cluster of recreational, passive or cognitively stimulating activities [18], [24], [25], [29]–[31], making it difficult to isolate a specific effect of this behavior. Consistent with our findings, TV viewing was positively associated with cognitive decline among Chinese subjects older than 55 years [28]. These authors suggested that engagement in such a passive activity could result from preclinical cognitive impairment. In line with this contention, the same research team also found that reading was associated with a reduced risk of cognitive impairment over a 5-year follow-up. In our models, however, reading was not associated with cognitive function.

### Cognitively stimulating leisure activities and cognitive function

The positive association between computer use and cognitive function is in line with previous research reporting a lower risk of dementia or cognitive decline among individuals with increased engagement in cognitively stimulating leisure activities [28]. Therefore, we can speculate that the mentally stimulating characteristics of some sedentary behaviors, such as computer use, may compensate for their relatively passive nature, regarding their impact on brain aging. The underlying mechanisms by which computer use may be related to cognitive function encompass the contribution of stimulating leisure activities to cognitive reserve on the one hand and the “use it or lose it” concept on the other hand. The cognitive reserve concept posits that some background factors (related to education, occupation, experience) may supply alternate capacities to cope with, or compensate for, neurologic damage thus delaying the clinical expression of dementia [50].

### Limitations

Some limitations of this analysis need to be mentioned. First, the single assessment of cognitive performance did not allow evaluating causal relationships. In addition, participants with preclinical symptoms of cognitive decline may have discontinued some types of leisure activity and reverse causality cannot be ruled out. It should be noted, however, that after exclusion of participants with possible cognitive impairment, the associations with computer use remained significant. Second, data on sedentary behaviors were derived from self-reports, which may have led to an underestimation of such behaviors and/or to a misclassification bias. In particular, we did not have information on the purposes of using the computer (eg, browsing the Internet, reading, playing games, blogging, etc.). Indeed, computer use could be an active or a passive occupation. Similarly, data on the types of reading material (textbooks, fiction, newspapers, comics, trade magazines, etc.) were not available which may partly explain the absence of an association with cognitive function. An objective measurement of sedentary behavior would require the use of movement counters such as accelerometers [51], although this method does not allow the assessment of the type of sedentary behavior performed. Third, we assessed sedentary behaviors only during leisure time because over three-quarters of the participants in our sample were retired. Finally, caution is needed when estimating the external validity of our findings as the participants were health-conscious, well-educated volunteers initially involved in a long-term nutritional intervention. This may have led to homogeneity in lifestyles and in cognitive profiles, reducing the probability of detecting significant associations. Furthermore, the analyzed sample may be considered as overselected because participants having all available data may have been particularly compliant, as shown by the comparison between included and excluded participants. These results should therefore be confirmed in other populations.

### Conclusion

In conclusion, the results of this study, carried out in healthy older persons, suggest that sedentary behaviors may not be equal in their impact on cognitive performance, depending on their level of cognitive stimulation. Computer use, a widespread occupation in technology-based societies, may be beneficial in maintaining cognitive function, especially verbal memory and executive functioning. Nonetheless, the potentially beneficial impact of computer use does not preclude a harmful effect in case of abuse and this specific effect should be investigated in ad-hoc studies. Widespread computer access, however, may become a public health challenge with regard to the maintenance of cognitive performance during aging. Further investigations in different settings, in particular long-term trials among healthy adults, are needed to confirm these findings.

## Supporting Information

Table S1 **Inclusion-Exclusion.** Comparison of characteristics of excluded and included participants, from the original SU.VI.MAX cohort (N = 13,017).
(DOCX)
