# Supplementary material for: Cross-Sectional and Longitudinal Associations of Different Sedentary Behaviors with Cognitive Performance in Older Adults
Source: PLoS One. 2012 Oct 17;7(10):e47831. doi: 10.1371/journal.pone.0047831 (PMC3474738; doi:10.1371/journal.pone.0047831)
Supplement: Table S1 — Inclusion-Exclusion. Comparison of characteristics of excluded and included participants, from the original SU.VI.MAX cohort (N = 13,017). (DOCX) [file pone.0047831.s001.docx]

Table S1: Comparison of characteristics of excluded and included participants, from the original SU.VI.MAX cohort (N=13,017)^a^

|  | Excluded participants | | Included participants | | P |
| --- | --- | --- | --- | --- | --- |
|  | *N* |  | *N* |  |  |
| Male, % | *10,438* | 35.6 | *2,579* | 55.3 | <.0001 |
| Age (1994), y ^c^ | *10,438* | 48.2 (6.5) | *2,579* | 52.2 (4.6) | <.0001 |
| BMI (1994) kg/m² ^c^ | *9,867* | 23.8 (3.7) | *2,543* | 23.9 (3.0) | <.0001 |
| Intervention group, % | *10,438* | 49.1 | *2,579* | 52.6 | 0.001 |
| Education, % ^c^ | *10,098* |  | *2,579* |  |  |
| Primary |  | 21.7 |  | 19.9 | 0.01 |
| Secondary |  | 37.4 |  | 40.7 |  |
| University |  | 40.9 |  | 39.4 |  |
| Smoking status, % ^c^ | *9,909* |  | *2,519* |  | <.0001 |
| Never-smokers |  | 45.5 |  | 49.9 |  |
| Former smokers |  | 36.7 |  | 40.3 |  |
| Current smokers |  | 17.8 |  | 9.8 |  |
| History of diabetes, % | *4,440* | 12.1 | *2,579* | 6.8 | <.0001 |
| History of hypertension, % | *5,419* | 59.9 | *2,579* | 58.7 | 0.30 |
| History of cardiovascular disease, % | *1,0162* | 3.2 | *2,579* | 4.7 | 0.0001 |
| Retired, %^d^ | *4,367* | 56.4 | *2,579* | 84.5 | <.0001 |
| Depression score (CES-D) ^d^ | *4,176* | 9.9 (8.5) | *2,579* | 8.5 (7.3) | <.0001 |
| Leisure-time physical activity, MET-h/week ^d^ | *3,033* | 22.4 (24.9 | *2,579* | 26.5 (27.2) | <.0001 |
| Watching TV, min/d ^d^ | *3,408* | 141.8 (87.9) | *2,579* | 147.2 (82.9) | 0.001 |
| Computer use, min/d ^d^ | *3,352* | 44.4 (63.5) | *2,579* | 48.8 (61.6) | <.0001 |
| Reading, min/d ^d^ | *3,428* | 69.6 (58.6) | *2,579* | 73.0 (54.9) | <.0001 |
| General health status, % ^c^ | *4,209* |  | *2,579* |  |  |
| Excellent |  | 11.5 |  | 10.4 | 0.01 |
| Good |  | 63.6 |  | 69.0 |  |
| Fair |  | 22.5 |  | 19.4 |  |
| Poor |  | 2.2 |  | 1.2 |  |
| Very poor |  | 0.2 |  | 0.04 |  |

^a^ Values are mean (SD) or % as appropriate except when otherwise noted

^b^ P is based on Kruskal-Wallis or chi² test, as appropriate

^c^ At baseline (1994)

^d^ At the SU.VI.MAX 2 examination (2007-2009)
